# Naples prognostic score, a novel prognostic score for patients with high- and intermediate-risk gastrointestinal stromal tumours after surgical resection

**DOI:** 10.1186/s12957-022-02526-0

**Published:** 2022-03-01

**Authors:** Hao Wu, Mengdi Fu, Xiaozhou Xie, Jianqiao Yang, Yang Liu, Fengying Du, Zhen Fang, Liang Shang, Leping Li

**Affiliations:** 1 Department of Gastroenterological Surgery, Shandong Provincial Hospital, Cheeloo College of Medicine, Shandong University, Jinan, 250021 Shandong China; 2 Department of Clinical Medicine, Cheeloo College of Medicine, Shandong University, Jinan, 250021 Shandong China; 3 Department of Gastroenterological Surgery, Shandong Provincial Hospital Affiliated to Shandong First Medical University, Jinan, 250021 Shandong China; 4 Department of Digestive Tumor Translational Medicine, Engineering Laboratory of Shandong Province, Shandong Provincial Hospital, Jinan, 250021 Shandong China; 5 Medical Science and Technology Innovation Center, Shandong First Medical University & Shandong Academy of Medical Sciences, Jinan, 250021 Shandong China

**Keywords:** Naples prognostic score, Gastrointestinal stromal tumours, Prognostic score, Prognosis, Progression-free survival, Overall survival

## Abstract

**Background:**

A novel multidimensional inflammatory and nutritional assessment system named the Naples prognostic score could serve as an independent prognostic indicator. However, its significance in patients with high- and intermediate-risk gastrointestinal stromal tumours remains unclear.

**Methods:**

We performed this retrospective cohort study based on a prospectively collected database of gastrointestinal stromal tumours (GISTs) between March 2010 and December 2019. The Kaplan–Meier method and log-rank test were used for survival analyses. Least absolute shrinkage and selection operator (LASSO) and Cox proportional hazards regression analysis was used for univariate and multivariate analyses. Time-dependent receiver operating characteristic curves were generated to evaluate the discriminatory ability of the prognostic scoring systems. Differences in the areas under the curve were further compared.

**Results:**

A total of 405 patients with regular follow-up were included and analysed in this study. Significant differences in progression-free survival and overall survival were observed between the groups (*P* < 0.001). Multivariate analysis demonstrated that the NPS was a significant predictor of poor progression-free survival (1 vs 0, *HR* = 4.622, *P* = 0.001; 2 vs 0, *HR* = 12.770, *P* < 0.001) and overall survival (2 vs 0, *HR* = 5.535, *P* = 0.002). Furthermore, time-dependent AUC analyses showed that the NPS was more accurate than other haematologic prognostic systems.

**Conclusions:**

The present study demonstrates that the NPS could independently predict disease progression and survival among patients with high- and intermediate-risk GISTs. The NPS might be regarded and applied as one of the most convenient and effective preoperative risk stratification tools in the future, which should be validated by large-scale multicentre prospective cohort studies.

**Supplementary Information:**

The online version contains supplementary material available at 10.1186/s12957-022-02526-0.

## Introduction

As the most common mesenchymal tumours of the digestive system with varying malignant potential, gastrointestinal stromal tumours (GISTs) have recently attracted increasing attention [1, 2]. Compared with the 7 per million population per year in the USA [3], the incidence of GISTs in China ranges from 4.3 to over 20 per million [4]. The widely recognized modified National Institutes of Health (NIH) classifications are used to predict the risk of recurrence by evaluating the primary tumour site, size, mitotic index, and tumour rupture [5]. Despite the great progress of surgical and adjuvant therapy over the past decade, the recurrence rate is still high, especially in high- and intermediate-risk patients [6]. In clinical practice, we found that the prognosis of patients with the same risk classification based on the modified NIH varies greatly. Therefore, it is necessary to develop an efficient classification system outside the modified NIH system to improve the accuracy of prognosis evaluation and make appropriate decisions.

It has been gradually recognized that the prognosis of patients is not only related to the characteristics of the tumour but also closely related to nutritional and immune-inflammatory status [7]. Recently, several haematological indices and scoring systems have been reported for GISTs, such as the neutrophil-to-lymphocyte ratio (NLR), platelet-to-lymphocyte ratio (PLR), prognostic nutritional index (PNI), systemic immune-inflammation index (SII), and Onodera’s prognostic nutritional index (OPNI) [8–14]. In addition, some comprehensive scoring systems, including the controlling nutritional status (CONUT), Glasgow Prognostic Score (GPS), and systemic inflammation score (SIS), have been reported to be applied to gastrointestinal tumours [15–17]. Most recently, a novel multidimensional comprehensive prognostic evaluation system, the Naples prognostic score (NPS), was established based on albumin, total cholesterol, NLR, and lymphocyte-monocyte ratio (LMR) although a prospective clinical trial [18]. This scoring system has been reported in oesophageal cancer, lung cancer, pancreatic cancer, colorectal cancer, endometrial cancer, osteosarcoma, and gastric cancer, which is of great significance [19–25]. However, the role of NPS in patients with GISTs remains unclear.

Therefore, our study was designed to investigate the value of NPS in patients with high- and intermediate-risk GISTs and compare its prognostic accuracy on progression-free survival and overall survival with that of other nutritional or inflammatory markers.

## Patients and methods

We performed this retrospective cohort study based on a prospectively collected database of GISTs at Shandong Provincial Hospital. All relevant procedures were approved by the Institutional Review Board (IRB). We designed this study in compliance with the Helsinki Declaration and the Strengthening the Reporting of Cohort Studies in Surgery statement. All protocols were approved by the Ethics Committee of Shandong University, China. The data were anonymous, and the requirement for informed consent was waived.

### Patients

**Fig. 1:**
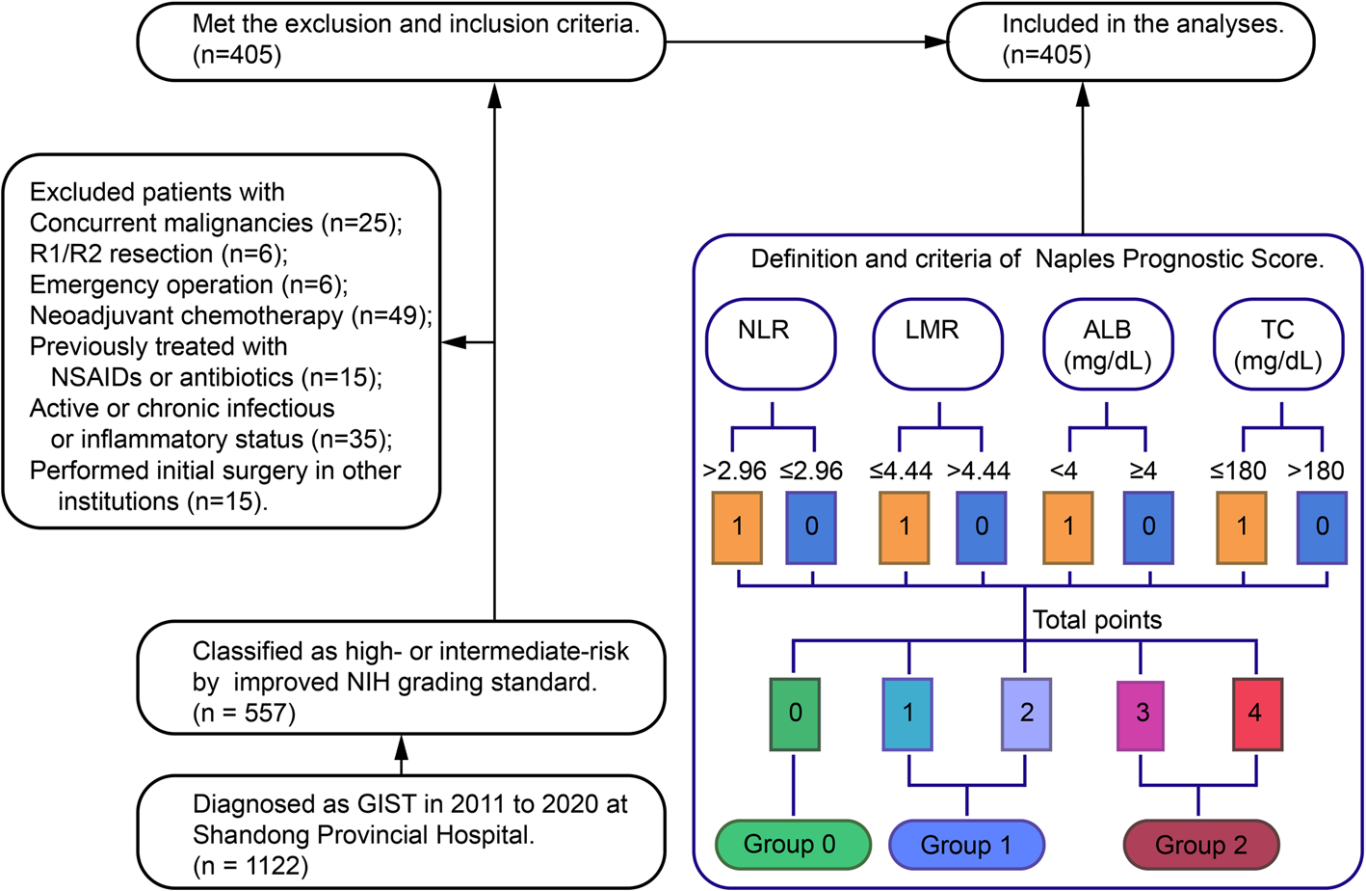
Flow chart of the analysis and definition and criteria of Naples prognostic score. GIST, gastrointestinal stromal tumours; NSAIDs, non-steroidal anti-inflammatory drugs; NLR, neutrophil-lymphocyte ratio; LMR, lymphocyte-monocyte ratio; ALB, albumin; TC, total cholesterol

A total of 1122 consecutive patients diagnosed with GIST and undergoing surgery between March 2010 and January 2020 at Shandong Provincial Hospital were initially pooled. Among them, 557 were classified as high or intermediate risk by the modified NIH grading standard (Fig. 1).

The inclusion criteria were as follows: (1) older than 18 years, (2) pathologically diagnosed with high- or intermediate-risk GIST, (3) primary localized GIST only, and (4) detailed and extractable medical data and laboratory results.

The exclusion criteria were as follows: (1) other concurrent malignancies; (2) R1/R2 resection or intraoperative tumour rupture; (3) emergency operation; (4) neoadjuvant therapy; (5) previous treatment with non-steroid anti-inflammatory drugs (NSAIDs) or antibiotics; (6) no routine blood examination before surgery; (7) active or chronic infectious or inflammatory status, such as haematological diseases, hepatopathy, and diseases of the immune system; (8) initial surgery performed in other institutions; and (9) blood transfusion within 3 months before surgery.

Finally, 346 patients with regular follow-up were included and analysed in this study. The follow-up was performed every 3 months for the first 3 years, then every 6 months up to 5 years, and then every year in the following years or until death. The latest follow-up date was December 2020. If the patient is lost to follow-up, the record from the last visit will be taken [26].

Follow-up assessment included physical examinations, blood tests, and imaging examinations. Endoscopic ultrasonography (EUS) is particularly important for judging the location and origin of the tumour and its relationship with surrounding organs. CT, especially enhanced CT, is the preferred imaging method for GIST, which helps to clarify the location, size, growth pattern, adjacent organs, blood supply, and distant metastasis of the tumour. MRI is of great significance for the evaluation of GIST in special parts such as the rectum, pelvic floor, or liver metastases. The re-examination tests include blood routine, liver and kidney function, and imaging examinations.

### Data collection

The clinicopathological characteristics of the patients were routinely collected from the GIST database, including age, sex, chief complaint, tumour location, tumour size, mitotic index, modified NIH risk category, surgical approach, intraoperative blood transfusion, perioperative complications, hospitalization time, postoperative imatinib, immunohistochemistry (IHC), and haematological indices. Routine blood tests were performed within 7 days before surgery. If there are multiple test results within the specified time, the average value will be taken. In the case of missing values in the covariates, such as albumin and total cholesterol, we assumed that these are missing at random (MAR), and we used multiple imputation models to provide valid and efficient estimates (Supplemental Fig. 1) [27]. Although there are other missing indicators, which did not affect the construction of the model, we still show them in the figure and fill them in.

When the serum albumin concentration was ≥ 4.0 mg/dl, total cholesterol was > 180 mg/dl, NLR was ≤ 2.96, or LMR was > 4.44, the sample was scored as 0; otherwise, it was scored as 1 (Fig. 1). NPS was defined as the sum of the aforementioned scores and divided into 3 groups. Patients with a score of ≥ 3 were assigned to group 2, patients with a score of 0 were assigned to group 0, and the remaining patients were assigned to group 1. The definitions and calculations of other nutritional- and immune-inflammatory-related indicators are listed in Supplemental Table 1.

### Statistical analysis

The primary outcome in this study was progression-free survival (PFS), which was defined as the interval between the operation date and the date of confirmed disease progression or death. The secondary outcome was overall survival (OS), which was calculated from the date of surgery until the date of death. Patients without the above events were censored at the date of the last follow-up.

Categorical variables were compared using Pearson’s chi-squared test or Fisher’s exact test according to the expected values. The Mann–Whitney *U*-test was utilized to analyse continuous variables, which are presented as medians and interquartile ranges (IQRs) or the mean ± standard deviation (SD) in some conditions. The Kaplan–Meier method and log-rank test were used to conduct survival analyses and evaluate the differences in survival times, respectively. The optimal cut-off values were determined by X-tile. The indexes were selected by least absolute shrinkage and selection operator (LASSO) Cox regression, which is suitable for analysing high-dimensional data with a limited sample size [28, 29]. Cox proportional hazards regression analysis was used for multivariate analyses. Hazard ratios (HRs) with their 95% confidence intervals (CIs) were also derived. Optimal cut-off values were established by calculating the Youden index (sensitivity + specificity − 1).

Time-dependent receiver operating characteristic (ROC) curves were generated to evaluate the discriminatory ability of the prognostic scoring systems. Differences in the areas under the curve (AUCs) were further compared. Higher AUC values indicate better predictive ability.

We used SPSS 22.0 (IBM SPSS Statistics, Armonk, NY: IBM Corp) and R software version 3.6.2 (The R Foundation for Statistical Computing, Vienna, Austria). Statistical significance was considered when *P* < 0.05.

## Results

### Relationships between clinicopathological characteristics and NPS

Of the 1122 consecutive patients, 557 patients met the inclusion criteria. After further excluding 152 patients, 405 patients were finally included. The relationships between clinicopathological characteristics and NPS are shown in Table 1. A total of 232 male and 173 female patients were included, and the mean age at diagnosis was 58.27 ± 10.75 years. The location of the tumour showed marginally significant differences among the three groups. More than half of the tumours were located in the stomach (229/405), the vast majority of which were located in the fundus (102/229) and body (115/229) of the stomach. Non-gastric GISTs were located at the jejunum (77/176), duodenum (40/176), ileum (33/176), and rectum (10/176). The conclusive stage of the 405 patients, according to the modified NIH classification, was intermediate risk in 139 patients and high risk in 266 patients. Among the 405 patients, 290 underwent open surgery and 115 underwent laparoscopy. Regarding short-term outcomes, postoperative complications were not significantly associated with the NPS groups.

**Table 1:** Association of Naples prognostic score and clinicopathological characteristics

Variables	Total *n*%	Naples prognostic score			*P*-value
		Group 0 *n*%	Group 1 *n*%	Group 2 *n*%	
All cases	405	46	204	155	
Age (years)					0.677
Mean ± SD	58.27 ± 10.75	58.17 ± 10.15	57.85 ± 10.72	58.86 ± 10.99	
BMI (kg/m^2^)					0.639
BMI < 18.5	16 (3.95)	0 (0.00)	8 (3.92)	8 (5.16)	
18.5 ≤ BMI < 25	225 (55.56)	26 (56.52)	114 (55.88)	85 (54.84)	
BMI ≥ 25	164 (40.49)	20 (43.48)	82 (40.20)	62 (40.00)	
Gender					**< 0.001**
Male	232 (57.28)	14 (30.43)	117 (57.35)	101 (65.16)	
Female	173 (42.72)	32 (69.57)	87 (42.65)	54 (34.84)	
Tumour location					0.090
Gastric	229 (56.54)	28 (60.87)	124 (60.78)	77 (49.68)	
Non-gastric	176 (43.46)	18 (39.13)	80 (39.22)	78 (50.32)	
Tumour size					0.286
Mean ± SD	9.291 ± 5.43	8.343 ± 4.02	9.172 ± 5.63	9.730 ± 5.51	
Mitotic index (per 50 HPF)					0.496
0–5	200 (49.38)	27 (58.70)	98 (48.04)	75 (48.39)	
6–10	107 (26.42)	12 (26.09)	57 (27.94)	38 (24.52)	
> 10	98 (24.20)	7 (15.22)	49 (24.02)	42 (27.10)	
NIH risk category					0.177
Intermediate	139 (34.32)	19 (41.30)	75 (36.76)	45 (29.03)	
High	266 (65.68)	27 (58.70)	129 (63.24)	110 (70.97)	
Surgical approach					**0.001**
Open	290 (71.60)	30 (65.22)	132 (64.71)	128 (82.58)	
Laparoscopy	115 (28.40)	16 (34.78)	72 (35.29)	27 (17.42)	
Multi-organ involved					0.896
Yes	51 (12.59)	6 (13.04)	27 (13.24)	18 (11.61)	
No	354 (87.41)	40 (86.96)	177 (86.76)	137 (88.39)	
Blood transfusion					**< 0.001**
Yes	115 (28.40)	6 (13.04)	45 (22.06)	64 (41.29)	
No	290 (71.60)	40 (86.96)	159 (77.94)	91 (58.71)	
Complications					0.680
Yes	182 (44.94)	18 (39.13)	92 (45.10)	72 (46.45)	
No	223 (55.06)	28 (60.87)	112 (54.90)	83 (53.55)	
Hospitalization time (days)					0.054
Mean ± SD	14.56 ± 5.82	13.22 ± 4.42	14.26 ± 5.29	15.35 ± 6.70	
Postoperative imatinib					0.852
Yes	183 (45.19)	20 (43.48)	95 (46.57)	68 (43.87)	
No	222 (54.81)	26 (56.52)	109 (53.43)	87 (56.13)	

Bold values indicate *P* < 0.05

*HPF* high power field, *SD* standard deviation, *IQR* interquartile range, *NIH* National Institutes of Health

Chief complaints and laboratory results were also analysed (Supplemental Table 2). The median albumin (ALB), total cholesterol (TC), neutrophil-to-lymphocyte ratio (NLR), and lymphocyte-to-monocyte ratio (LMR) were 39.1 g/l (IQR, 35.6–42.2), 4.53 mmol/l (IQR, 3.89–5.41), 2.32 (IQR, 1.60–3.61), and 3.85 (IQR, 2.57–5.51), respectively. Statistical analysis showed that sex, surgical approach, interoperative, and blood transfusion of the patients in NPS groups 0, 1, and 2 were significantly different (*P* < 0.001, *P* = 0.001, and *P* < 0.001, respectively). Age, body mass index (BMI), tumour location, tumour size, mitotic index (per 50 HPF), modified NIH risk category, multi-organ involvement, perioperative complications, and postoperative imatinib did not show significant differences among the 3 groups. There were no significant differences in abdominal pain, abdominal distention, haematemesis, or most of the immunohistochemistry (IHC) results, while haematochezia, incidental findings, and most blood indicators were significantly different.

### Progression-free survival and overall survival based on NPS

The median follow-up period was 1561 (IQR, 1024–2200) days. The 1-, 3-, and 5-year PFS rates were 94.32%, 72.51%, and 51.62%, while the 1-, 3-, and 5-year OS rates were 96.53%, 89.08%, and 70.10%, respectively.

**Fig. 2:**
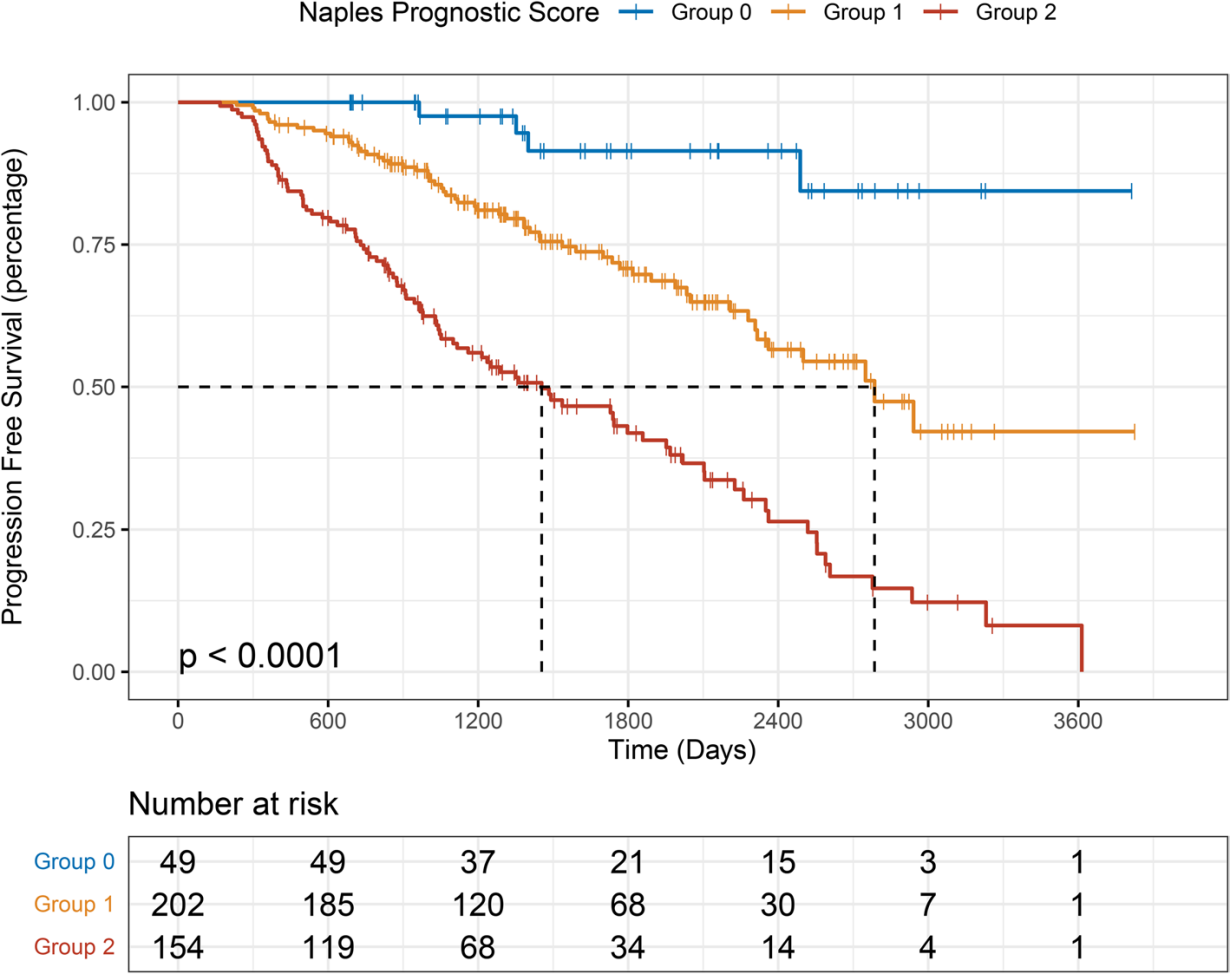
Kaplan–Meier survival analysis of progression-free survival

**Fig. 3:**
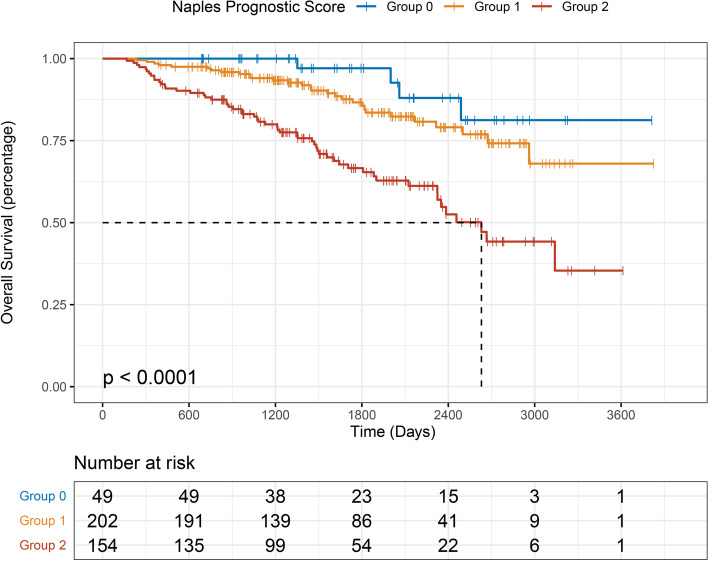
Kaplan–Meier survival analysis of overall survival

For PFS, significant differences were observed among the three groups, and the lower NPS group was significantly associated with longer PFS (*P* < 0.001; Fig. 2). The median PFS time of each NPS group was 1671.5 days in group 0, 1357 days in group 1, and 1041 days in group 2. Additionally, the median OS time of each NPS group was 1722.5 days in group 0, 1590 days in group 1, and 1501 days in group 2. Significant survival differences were also observed (*P* < 0.001; Fig. 3).

Thereafter, we conducted a subgroup analysis and found that regardless of whether postoperative adjuvant chemotherapy was performed, regardless of whether there was multiple organ invasion, and regardless of the modified NIH classification, mitotic index, tumour size, and tumour location, significant differences were observed in the NPS groups.

### Multivariate analyses of prognostic factors

In this study, the LASSO-Cox regression analysis was performed, and several clinicopathological factors were analysed to determine independent prognostic factors of PFS (Table 2) (Supplemental Fig. 2, A-B). Through the LASSO method, we identified the following significant prognostic factors for PFS: age, sex, BMI, tumour location, tumour size, mitotic index, surgical approach, multi-organ involvement, intraoperative blood transfusion, postoperative imatinib, and NPS group. In the multivariate analysis, tumour location (*HR* = 0.523, 95% *CI* = 0.372–0.736, *P* < 0.001), tumour size (*HR* = 1.841, 95% *CI* = 1.255–2.700, *P* = 0.002), mitotic index (*HR* =1.752, 95% *CI* = 1.186–2.589, *P* = 0.005), multi-organ involvement (*HR* = 1.868, 95% *CI* = 1.208–2.889, *P* = 0.005), postoperative imatinib (*HR* = 0.493, 95% *CI* = 0.350–0.696, *P* < 0.001), and NPS group (1 vs 0, *HR* = 4.622, 95% *CI* = 1.807–11.820, *P* = 0.001; 2 vs 0, *HR* = 12.770, 95% *CI* = 4.932–33.059, *P* < 0.001) were identified as independent prognostic factors.

**Table 2:** Multivariate of the clinicopathological factors for progression-free survival and overall survival

Characteristics	Progression-free survival		Overall survival	
	*HR* (95% *CI*)	*P*-value	*HR* (95% *CI*)	*P*-value
Age (years)		0.380		
≤ 65	Reference			
> 65	0.855 (0.602–1.213)			
Gender		0.606		
Female	Reference			
Male	1.096 (0.774–1.551)			
BMI (kg/m^2^)		0.599		0.613
BMI < 18.5	Reference		Reference	
18.5 ≤ BMI < 25	0.756 (0.345–1.658)	0.486	0.836 (0.339–2.062)	0.697
BMI ≥ 25	0.680 (0.305–1.518)	0.347	0.680 (0.251–1.799)	0.428
Tumour location		**< 0.001**		**< 0.001**
Non-gastric	Reference		Reference	
Gastric	0.523 (0.372–0.736)		0.409 (0.250–0.669)	
Tumour size (cm)		**0.002**		**0.018**
≤ 10	Reference		Reference	
> 10	1.841 (1.255–2.700)		1.889 (1.113–3.206)	
Mitotic index (per 50 HPF)		**0.005**		0.115
0–5	Reference		Reference	
6–10	0.891 (0.582–1.365)	0.595	1.103 (0.609–1.995)	0.747
> 10	1.752 (1.186–2.589)	**0.005**	1.749 (1.017–3.007)	**0.043**
Surgical approach		0.209		0.482
Laparoscopy	Reference		Reference	
Open	1.413 (0.824–2.426)		1.355 (0.581–3.159)	
Multi-organ involved		**0.005**		0.173
No	Reference		Reference	
Yes	1.868 (1.208–2.889)		1.473 (0.844–2.570)	
Blood transfusion		0.865		0.655
No	Reference		Reference	
Yes	1.032 (0.721–1.476)		1.118 (0.686–1.821)	
Postoperative imatinib		**< 0.001**		**< 0.001**
No	Reference		Reference	
Yes	0.493 (0.350–0.696)		0.405 (0.246–0.669)	
NPS group		**< 0.001**		**< 0.001**
0	Reference		Reference	
1	4.622 (1.807–11.820)	**0.001**	1.945 (0.668–5.660)	0.222
2	12.770 (4.932–33.059)	**< 0.001**	5.535 (1.923–15.929)	**0.002**

Bold values indicate *P* < 0.05

*HPF* high power field, *NIH* National Institutes of Health, *NPS* Naples prognostic score

In the LASSO model of the entire cohort (Supplemental Fig. 2, C-D), OS was significantly related to BMI, tumour location, tumour size, mitotic index, surgical approach, multi-organ involvement, intraoperative blood transfusion, postoperative imatinib, and NPS group. Finally, multivariable analysis determined that tumour location (*HR* = 0.409, *95% CI* = 0.250–0.669, *P* < 0.001), postoperative imatinib (*HR* = 0.405, 95% *CI* = 0.246–0.669, *P* < 0.001), and NPS group (2 vs 0, *HR* = 5.535, 95% *CI* = 1.923–15.929, *P* < 0.001) were significant.

### Prognostic value of the NPS and other parameters

**Fig. 4:**
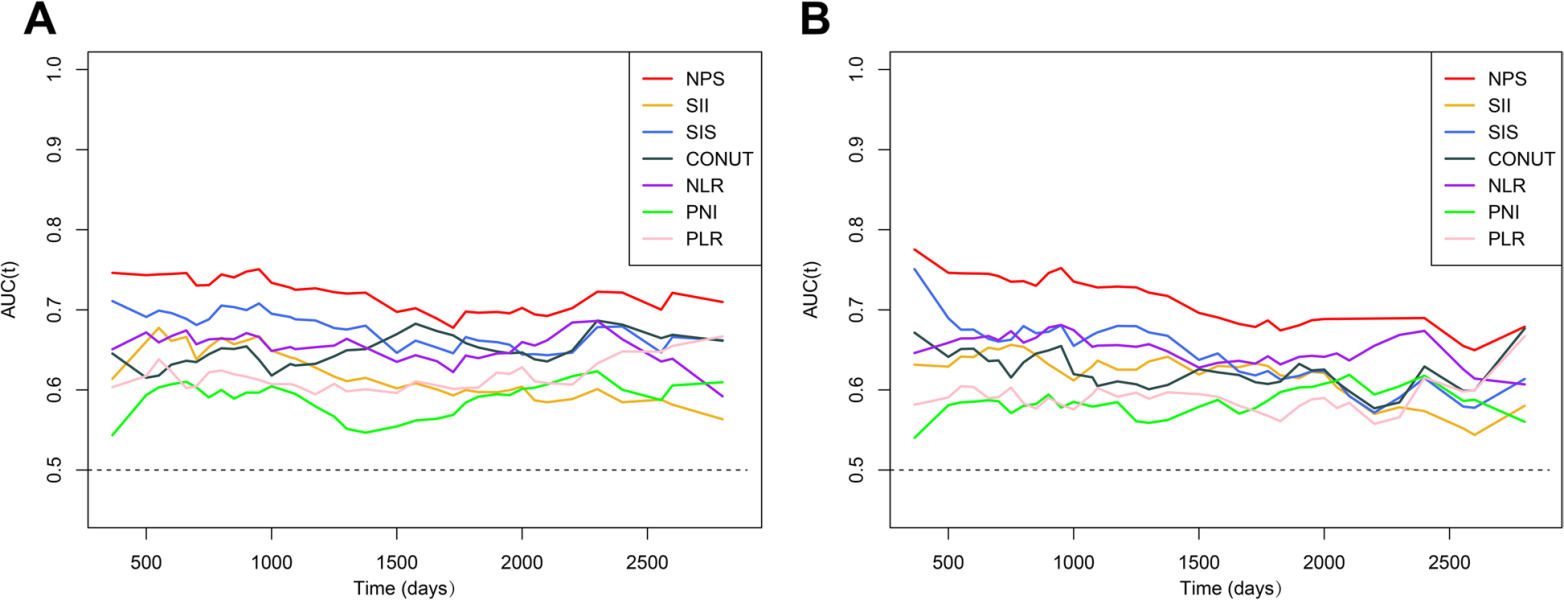
The time-dependent AUC curve analyses of prediction models. **A** Progression-free survival. **B** Overall survival. The horizontal axis represents the follow-up time, and the vertical axis represents the estimated AUC for survival at a specific time of interest. NPS, Naples prognostic score; SII, systemic immune-inflammation index; SIS, systemic inflammation score; CONUT, controlling nutritional status score; NLR, neutrophil-lymphocyte ratio; PNI, prognostic nutrition index; PLR, platelet-lymphocyte ratio

We compared the prognostic impact of the NPS with that of other evaluated haematologic biomarkers by time-dependent AUC analyses, which confirmed that the NPS had stronger discriminatory power and higher clinical values than any others, for both PFS and OS (Fig. 4).

Taking 3-year PFS as an example, the areas under the curve of the NPS, SII, SIS, CONUT, NLR, PNI, and PLR were 0.729, 0.639, 0.688, 0.631, 0.651, 0.595, and 0.606, respectively. The NPS was slightly better than the SIS and significantly more accurate than the rest of the above indices in predicting 3-year PFS. The NPS also has certain advantages at 1 and 7 years in predicting disease progression, but the advantages were not significant (Supplemental Table 3).

## Discussion

Abundant evidence suggests that systemic inflammatory reactions in the tumour microenvironment facilitate tumour cell proliferation, angiogenesis, metastasis, and anticancer drug resistance and even disrupt antitumour immunity [30, 31]. Numerous prognostic systems based on systemic inflammatory and nutritional indicators have been developed for various types of tumours. However, a single marker is too easily influenced when calculating the cut-off value arbitrarily, thus greatly affecting its clinical application. Therefore, multidimensional prognostic evaluation systems, such as the NPS, which covers the joint effects of NLR, LMR, ALB, and TC, may be more stable and accurate. In the present study, the value of the NPS in patients with high- and intermediate-risk GISTs was thoroughly assessed. The NPS was certified to be strongly correlated with both progression-free survival and overall survival and also showed superior accuracy to other previously reported scoring systems.

According to the existing literature, the NPS is believed to reflect different severities of the systemic inflammatory response and malnutrition status, and the underlying biological mechanisms may be as follows.

First, a meta-analysis showed that a low albumin level could be an adverse factor with a significant PFS advantage for high-dose imatinib [32]. Patients with low albumin levels are more likely to have a lower surgical tolerability due to a lack of immune defence capability [33]. Albumin levels could reflect systemic inflammation because they are affected by proinflammatory substances [34].

Second, plasma cholesterol and albumin levels have been applied to assess clinical nutritional status. In addition, the membranous fluidity of cells and the capacity of transmembrane signal transmission were severely affected by hypocholesterolaemia [35]. Therefore, immunocompetent cells fail to destroy cancer cells [36]. In addition, lipid metabolism may be related to the proliferation and metastasis of GISTs [37, 38].

Third, a large number of monocyte-derived cells were observed in GISTs, which points to a possible ‘symbiotic relationship’ between the tumour and the local immune cells [39]. Tumour-associated macrophages (TAMs), differentiated from monocytes in some cases, could promote proliferation and metastasis [40]. Under inflammatory conditions, highly represented monocytes could disseminate tumour cells through circulation [41]. Thus, the elevated number of peripheral monocytes may reveal a high cancer burden.

Fourth, neutrophils, as one of the most important mediators in the immune system, could also be mobilized and recruited to tumours. Aberrant accumulation of neutrophils is often associated with poor prognosis due to cell arrest and tissue disruption [42]. Neutrophils can also produce a variety of proteins, such as MMPs, to promote cancer migration and invasion [43].

Last, lymphocytes, which are fundamental for the immune system, also play a vital role in immune editing and surveillance and could inhibit the progression and metastasis of cancer [44]. Therefore, both NLR and LMR could reflect inflammation and participate in the immune response to tumours.

In summary, the NPS established on the basis of the above 5 indicators could reflect both inflammatory and nutritional states more precisely to assist surgeons in the achievement of personalized therapy for patients.

However, the creator of the scoring system said that patients with the worst Naples prognostic score experienced more postoperative complications, and our study does not support this conclusion [18]. Similarly, two of the subsequently published articles had the same conclusions as ours [20, 21], and the remaining articles did not list data related to postoperative morbidity [19, 22–25]. This finding may be related to the careful supervision and continuous maintenance of the patient’s immune-nutritional status by attending physicians during hospitalization. In addition, we found differences in the length of hospitalization and hypothesized that the operation method and difficulty might be the main influencing factors. With the advancement of medical technology and the improvement of nutrition theory, the predictive strength of NPS for postoperative complications is gradually being attenuated.

Several haematological indices and scoring systems that reflect nutritional and inflammatory status have been reported previously. We made a direct comparison among them, and the results supported that the NPS is the best due to the high accuracy and convenient calculation. As shown in the comparison of time-dependent AUCs, the NPS was a significantly more accurate predictive system for predicting 3-year PFS and maintained a leading position for all PFS times. However, in predicting 1-year and 7-year PFS, the advantage was not significant enough, which may be related to the good prognosis of GISTs and short follow-up. In addition, in terms of OS, there was only a significant difference when comparing the NPS groups 0 and 1 and group 2. Since there are no widely accepted models yet, the presented study also has unprecedented value.

GISTs, as a low incidence gastrointestinal tumour, are gradually gaining the attention of oncologists, surgeons, and gastroenterologists. In addition to studies on the clinical characteristics and prognosis of gastrointestinal stromal tumours, such as the long-term survival of patients with gastrointestinal stromal tumours diagnosed after malignant tumours [45], the whole-process management of gastrointestinal stromal tumours is gradually enriched and improved, such as preoperative adjuvant therapy for locally advanced and recurrent/metastatic gastrointestinal stromal tumours [46]. However, in clinical diagnosis and treatment, concise and efficient prognostic evaluation criteria are still the most concerned issue for clinicians; we calculated and compared all the commonly used prognostic markers and found that the Naples score may be the most practical comprehensive evaluation system.

Our study has many highlights. This is the first time this novel scoring system has been used for GIST prognostic evaluation, and its accuracy surpasses that of other indicators. In addition, such a large-scale study of high- and intermediate-risk GISTs is rare worldwide. Thus, our findings are of great value for surgeons to preoperatively judge the survival benefit of patients and to formulate the most appropriate treatment strategy. Finally, we also proved that the score can be used as a prediction indicator for both OS and PFS. For PFS, there were significant differences in survival curves in multiple subgroups. In recent years, with the gradual acceptance of neoadjuvant therapy, we have also preliminarily found similar trends in such patients, but further follow-up and analyses are still needed.

This study still has some limitations. Despite strict screening in the prospectively established database, selection bias still exists due to the retrospective nature of the study. The relatively small sample size of a single centre might also limit the significance of the study. In addition, there are some shortcomings in our data collection. Postoperative complications were not strictly recorded according to the Clavien–Dindo grade, even though a significant difference was not observed. Similarly, several inflammatory markers, such as C-reactive protein, fibrinogen, and cytokines, were not assessed adequately and need to be collected and evaluated in the future. Whether the postoperative NPS score can also play a predictive role and the clinical significance of the changes in the trend still need to be further considered and interpreted. Of course, what cannot be ignored is that although we deal with missing values and lost data as much as possible, it is still difficult to avoid bias.

## Conclusions

The present study provides the first evidence that the NPS independently predicts disease progression and survival among patients with high- and intermediate-risk GISTs. The NPS might be regarded and applied as one of the most convenient and effective preoperative risk stratification models in the future, which should be validated by large-scale multicentre prospective cohort studies.

## Supplementary Information

**Additional file 1: Table S1.** Definition and calculation of nutritional and immune-inflammatory related indicators.

**Additional file 2: Table S2.** Association of Naples prognostic score and supplementary clinicopathological characteristics. Bold values indicate P<0.05; *median (IQR). IHC: Immunohistochemistry; WBC: White Blood Cell; RBC: Red Blood Cell; HGB: Hemoglobin; PLT: Platelet; LYM: Lymphocyte; MON: Monocyte; NEU: Neutrophil; EO: Eosinophil; BASO: Basophil; AST: Aspartate Aminotransferase; ALT: Alanine Aminotransferase; PA: Prealbumin; ALB: Albumin; GLO: Globulin; TC: Total Cholesterol; FIB: Fibrinogen; NLR: Neutrophil-Lymphocyte Ratio; LMR: Lymphocyte-Monocyte Ratio; NA: Not Available.

**Additional file 3: Table S3.** Comparison of time-dependent AUC curve analyses of prediction models for PFS. PFS: Progression-free Survival; NPS: Naples Prognostic Score; SII: Systemic Immune-inflammation index; SIS: Systemic Inflammation Score; CONUT: Controlling Nutritional Status Score; NLR: Neutrophil-Lymphocyte Ratio; PNI: Prognostic Nutrition Index; PLR: Platelet-Lymphocyte Ratio.

**Additional file 4: Figure S1.** Missing values and multiple imputation.

**Additional file 5: Figure S2.** Clinical indicators selection using the LASSO Cox regression model. LASSO coefficients of total clinical indicators for PFS (A) and OS (C). Nonzero coefficients were determined based on the optimal log (lambda); log (lambda) and partial likelihood deviance were shown for PFS (B) and OS (D), the dotted line is displayed at the minimum log (lambda) represents the optimal number of predictors.

## Data Availability

Not applicable.

## Abbreviations

GIST: Gastrointestinal stromal tumour
HPF: High power field
SD: Standard deviation
LASSO: Least absolute shrinkage and selection operator
IQR: Interquartile range
CI: Confidence interval
HR: Hazard ratios
NIH: National Institutes of Health
BMI: Body mass index
NSAIDs: Non-steroidal anti-inflammatory drugs
PFS: Progression-free survival
OS: Overall survival
NPS: Naples prognostic score
SII: Systemic immune-inflammation index
SIS: Systemic inflammation score
CONUT: Controlling Nutritional Status Score
PNI: Prognostic Nutrition Index
PLR: Platelet-lymphocyte ratio
IHC: Immunohistochemistry
WBC: White blood cell
RBC: Red blood cell
HGB: Haemoglobin
PLT: Platelet
LYM: Lymphocyte
MON: Monocyte
NEU: Neutrophil
EO: Eosinophil
BASO: Basophil
AST: Aspartate aminotransferase
ALT: Alanine aminotransferase
PA: Prealbumin
ALB: Albumin
GLO: Globulin
TC: Total cholesterol
FIB: Fibrinogen
NLR: Neutrophil-lymphocyte ratio
LMR: Lymphocyte-monocyte ratio
NA: Not available
