# Supplementary material for: Naples prognostic score, a novel prognostic score for patients with high- and intermediate-risk gastrointestinal stromal tumours after surgical resection
Source: World J Surg Oncol. 2022 Mar 1;20:63. doi: 10.1186/s12957-022-02526-0 (PMC8886834; doi:10.1186/s12957-022-02526-0)
Supplement: Supplementary file 1 — Additional file 1: Table S1. Definition and calculation of nutritional and immune-inflammatory related indicators. [file 12957_2022_2526_MOESM1_ESM.docx]

| Abbreviation | Definition | Criteria |
| --- | --- | --- |
| NPS | Naples prognostic score | When the serum albumin concentration≥4.0 mg/dl, total cholesterol >180 mg/dl, NLR≤2.96 or LMR>4.44, then each be scored as 0, otherwise, which would be scored as 1 (Figure 1). NPS was defined as the sum of the aforementioned scores and divided into 3 groups. Patients with a score of ≥3 were assigned to group 2, patients with a score of 0 were assigned to group 0, rest of the patients were assigned to group 1. |
| NLR | Neutrophil-lymphocyte ratio | Neutrophil/lymphocyte |
| LMR | lymphocyte-monocyte ratio | Lymphocyte/monocyte |
| PLR | Platelet-lymphocyte ratio | Platelet/lymphocyte |
| SIS | Systemic inflammation score | the scores were 2 for cases having serum Alb concentration < 40 g/L and LMR <4.44; the scores were 1 for cases having serum Alb concentration ≥ 40 g/l or LMR ≥ 4.44; and the scores were 0 for cases having both serum Alb concentration ≥ 40 g/l and LMR ≥ 4.44. |
| SII | Systemic inflammation index | neutrophil × monocyte /lymphocyte |
| GPS | Glasgow Prognostic Score | patients with both an elevated CRP (>10 mg/L) and hypoalbuminemia (<35 g/L) were allocated a GPS score of 2. Patients in whom only one of these biochemical abnormalities was present were allocated a GPS score of 1. Patients in whom neither of these abnormalities was present were allocated a GPS score of 0. |
| mGPS | Modified Glasgow prognostic score | GPS was modified such that patients with hypoalbuminemia were assigned a mGPS score of 0 in the absence of an elevated CRP concentration. |
| PNI | Prognostic nutrition index | 10 × serum albumin level (g/dl) + 0.005 × total lymphocyte count (per mm2). |
| CONUT | Controlling nutritional status score | CONUT scores were calculated using serum albumin concentrations, peripheral lymphocyte counts, and total cholesterol concentrations. Albumin concentrations > 3.5 g/  dL, 3.0–3.49 g/dL, 2.5–2.99 g/dL, and < 2.5 g/dL were  scored as 0, 2, 4, 6, respectively. Total lymphocyte  counts > 1600/mm^3^, 1200–1599/mm^3^, 800–1199/mm^3^,  and < 800/mm3 were scored as 0, 1, 2, 3, respectively.  Total cholesterol concentrations > 180 mg/dL,  140–179 mg/dL, 100–139 mg/dL, and < 100 mg/dL were  scored as 0, 1, 2, 3, respectively. The CONUT score is the  summation of the three scores. |

**Supplemental Table 1**

**Definition and calculation of nutritional and immune-inflammatory related indicators.**
