# Supplementary material for: Naples prognostic score, a novel prognostic score for patients with high- and intermediate-risk gastrointestinal stromal tumours after surgical resection
Source: World J Surg Oncol. 2022 Mar 1;20:63. doi: 10.1186/s12957-022-02526-0 (PMC8886834; doi:10.1186/s12957-022-02526-0)
Supplement: Supplementary file 2 — Additional file 2: Table S2. Association of Naples prognostic score and supplementary clinicopathological characteristics. Bold values indicate P<0.05; *median (IQR). IHC: Immunohistochemistry; WBC: White Blood Cell; RBC: Red Blood Cell; HGB: Hemoglobin; PLT: Platelet; LYM: Lymphocyte; MON: Monocyte; NEU: Neutrophil; EO: Eosinophil; BASO: Basophil; AST: Aspartate Aminotransferase; ALT: Alanine Aminotransferase; PA: Prealbumin; ALB: Albumin; GLO: Globulin; TC: Total Cholesterol; FIB: Fibrinogen; NLR: Neutrophil-Lymphocyte Ratio; LMR: Lymphocyte-Monocyte Ratio; NA: Not Available. [file 12957_2022_2526_MOESM2_ESM.docx]

| Variables | Total n% | Naples prognostic score | | | *P*-value |
| --- | --- | --- | --- | --- | --- |
|  |  | Group 0 n% | Group 1 n% | Group 2 n% |  |
| All cases | 405 | 46 | 204 | 155 |  |
| Chief complaint |  |  |  |  |  |
| Abdominal pain | 124/405 (36.30) | 14/46 (30.43) | 70/204 (34.31) | 63/155 (40.65) | 0.132 |
| Abdominal distention | 47/405 (13.58) | 7/46 (15.22) | 26/204 (12.75) | 22/155 (14.19) | 0.871 |
| Hematemesis | 19/405 (5.43) | 1/46 (2.17) | 9/204 (4.41) | 12/155 (7.74) | 0.226 |
| Hematochezia | 69/405 (20.74) | 2/46 (4.35) | 36/204 (17.65) | 46/155 (29.68) | **<0.001** |
| Medical examination | 101/405 (27.65) | 20/46 (43.48) | 63/204 (30.88) | 29/155 (18.71) | **0.001** |
| Other | 25/405 (7.16) | 4/46 (8.70) | 15/204 (7.35) | 10/155 (6.45) | 0.864 |
| IHC |  |  |  |  |  |
| CD117 (+) | 395/401 (98.50) | 45/46 (97.83) | 200/202 (99.01) | 150/153 (98.04) | 0.698 |
| CD34 (+) | 344/398 (86.43) | 41/46 (89.13) | 176/199 (88.44) | 127/153 (83.01) | 0.286 |
| DOG-1 (+) | 389/398 (97.74) | 45/46 (97.83) | 193/200 (96.50) | 151/152 (99.34) | 0.206 |
| SMA (+) | 78/381 (20.47) | 7/45 (15.56) | 36/191 (18.85) | 35/145 (24.14) | 0.337 |
| S-100 (+) | 16/378 (4.23) | 1/44 (2.27) | 7/189 (3.70) | 8/145 (5.52) | 0.566 |
| Desmin (+) | 7/130 (5.38) | 1/15 (6.67) | 3/70 (4.29) | 3/45 (6.67) | 0.835 |
| Ki-67* | 5 (3-10) | 4 (3-6.5) | 5 (3-10) | 7 (4-10) | 0.100 |
| Blood indicators |  |  |  |  |  |
| WBC,10^9^/L * | 5.67 (4.67-7.14) | 5.08 (4.28-6.11) | 5.31 (4.54-6.30) | 6.64 (5.36-9.26) | **<0.001** |
| RBC, 10^12^/L * | 4.23 (3.67-4.66) | 4.45 (4.11-4.82) | 4.40 (3.92-4.71) | 3.88 (3.27-4.39) | **<0.001** |
| HGB, g/L* | 122 (96-138) | 136 (125-149) | 129 (103-143) | 101 (83-126) | **<0.001** |
| PLT, 10^9^/L * | 243 (199-312) | 235 (191-275) | 237 (196-303) | 263 (206-331) | **0.037** |
| LYM, 10^9^/L * | 1.46 (1.14-1.83) | 1.82 (1.44-2.42) | 1.58 (1.31-1.93) | 1.17 (0.87-1.47) | **<0.001** |
| MON, 10^9^/L * | 0.40 (0.29-0.52) | 0.31 (0.25-0.38) | 0.38 (0.27-0.47) | 0.47 (0.35-0.69) | **<0.001** |
| NEU, 10^9^/L * | 3.43 (2.61-4.82) | 2.67 (2.32-3.17) | 3.03 (2.34-3.91) | 4.77 (3.53-7.53) | **<0.001** |
| EO, 10^9^/L * | 0.08 (0.04-0.14) | 0.08 (0.04-0.13) | 0.09 (0.06-0.17) | 0.07 (0.03-0.12) | **0.028** |
| BASO, 10^9^/L * | 0.01 (0.01-0.02) | 0.02 (0.01-0.03) | 0.02 (0.01-0.03) | 0.01 (0.01-0.02) | 0.845 |
| AST, U/L* | 19 (16-24) | 21 (19-23) | 19 (16-23) | 19 (15-27) | **0.016** |
| ALT, U/L * | 15 (11-21) | 15.5 (11-23) | 14 (12-20) | 15 (11-20) | 0.090 |
| ALP, U/L * | 79 (65-99) | 79.5 (66-100) | 82 (69-99) | 77 (60-98) | 0.750 |
| PA, g/L* | 204 (100-248) | 247 (218-282) | 226 (183-259) | 152 (121-199) | **<0.001** |
| ALB, g/L* | 39.1 (35.6-42.2) | 43.8 (42.2-45.5) | 40.3 (38.0-42.8) | 35.8 (32.7-38.1) | **<0.001** |
| GLO, g/L * | 26.7 (23.8-30.1) | 29.2 (26.3-31.4) | 27.0 (24.6-30.2) | 25.2 (22.0-28.7) | **<0.001** |
| TC, mmol/L* | 4.53 (3.89-5.41) | 5.83 (5.26-6.51) | 4.89 (4.26-5.58) | 3.95 (3.34-4.43) | **<0.001** |
| FIB, g/l* | 2.97 (2.44-3.81) | 2.74 (2.36-3.27) | 2.89 (2.51-3.52) | 3.27 (2.46-4.64) | **<0.001** |
| NLR* | 2.32 (1.60-3.61) | 1.53 (1.21-2.03) | 1.95 (1.48-2.45) | 4.08 (3.09-6.74) | **<0.001** |
| LMR* | 3.85 (2.57-5.51) | 5.70 (5.02-7.38) | 4.43 (3.33-5.99) | 2.50 (1.79-3.46) | **<0.001** |
| Blood type |  |  |  |  | / |
| A | 99/405 (24.44) | 10/46 (21.74) | 51/204 (25.00) | 38/155 (24.52) |  |
| B | 142/405 (35.06) | 14/46 (30.43) | 70/204 (34.31) | 58/155 (37.42) |  |
| O | 115/405 (28.40) | 16/46 (34.78) | 56/204 (27.45) | 43/155 (27.74) |  |
| AB | 45/405 (11.11) | 6/46 (13.04) | 24/204 (11.76) | 15/155 (9.68) |  |
| NA | 4/405 (0.99) | 0/46 (0.00) | 3/204 (1.47) | 1/155 (0.65) |  |

**Supplemental Table 2**

**Association of Naples prognostic score and supplementary clinicopathological characteristics.**

Bold values indicate P<0.05; *median (IQR).

IHC: Immunohistochemistry; WBC: White Blood Cell; RBC: Red Blood Cell; HGB: Hemoglobin; PLT: Platelet; LYM: Lymphocyte; MON: Monocyte; NEU: Neutrophil; EO: Eosinophil; BASO: Basophil; AST: Aspartate Aminotransferase; ALT: Alanine Aminotransferase; PA: Prealbumin; ALB: Albumin; GLO: Globulin; TC: Total Cholesterol; FIB: Fibrinogen; NLR: Neutrophil-Lymphocyte Ratio; LMR: Lymphocyte-Monocyte Ratio; NA: Not Available.
