# Supplementary material for: Naples prognostic score, a novel prognostic score for patients with high- and intermediate-risk gastrointestinal stromal tumours after surgical resection
Source: World J Surg Oncol. 2022 Mar 1;20:63. doi: 10.1186/s12957-022-02526-0 (PMC8886834; doi:10.1186/s12957-022-02526-0)
Supplement: Supplementary file 3 — Additional file 3: Table S3. Comparison of time-dependent AUC curve analyses of prediction models for PFS. PFS: Progression-free Survival; NPS: Naples Prognostic Score; SII: Systemic Immune-inflammation index; SIS: Systemic Inflammation Score; CONUT: Controlling Nutritional Status Score; NLR: Neutrophil-Lymphocyte Ratio; PNI: Prognostic Nutrition Index; PLR: Platelet-Lymphocyte Ratio. [file 12957_2022_2526_MOESM3_ESM.docx]

| AUC (t) | 1-year | 3-years | 5-years | 7-years |
| --- | --- | --- | --- | --- |
| NPS | 0.746 (Reference) | 0.725 (Reference) | 0.696 (Reference) | 0.700 (Reference) |
| SII | 0.613 (*P* = 0.075) | 0.639 (*P* = 0.011) | 0.597 (*P =* 0.006) | 0.588 (*P =* 0.013) |
| SIS | 0.710 (*P* = 0.514) | 0.688 (*P* = 0.191) | 0.662 (*P =* 0.268) | 0.647 (*P =* 0.190) |
| CONUT | 0.645 (*P* = 0.203) | 0.631 (*P* = 0.004) | 0.635 (*P =* 0.382) | 0.664 (*P* = 0.742) |
| NLR | 0.651 (*P* = 0.007) | 0.651 (*P* = 0.014) | 0.640 (*P =* 0.066) | 0.636 (*P =* 0.200) |
| PNI | 0.543 (*P* = 0.027) | 0.595 (*P* < 0.001) | 0.591 (*P =* 0.025) | 0.587 (*P* = 0.117) |
| PLR | 0.603 (*P* = 0.007) | 0.606 (*P =* 0.001) | 0.603 (*P =* 0.023) | 0.648 (*P =* 0.569) |

**Supplemental Table 3**

**Comparison of time-dependent AUC curve analyses of prediction models for PFS.**

PFS: Progression-free Survival; NPS: Naples Prognostic Score; SII: Systemic Immune-inflammation index; SIS: Systemic Inflammation Score; CONUT: Controlling Nutritional Status Score; NLR: Neutrophil-Lymphocyte Ratio; PNI: Prognostic Nutrition Index; PLR: Platelet-Lymphocyte Ratio.
