# Supplementary figures and images for: Naples prognostic score, a novel prognostic score for patients with high- and intermediate-risk gastrointestinal stromal tumours after surgical resection
Source: World J Surg Oncol. 2022 Mar 1;20:63. doi: 10.1186/s12957-022-02526-0 (PMC8886834; doi:10.1186/s12957-022-02526-0)

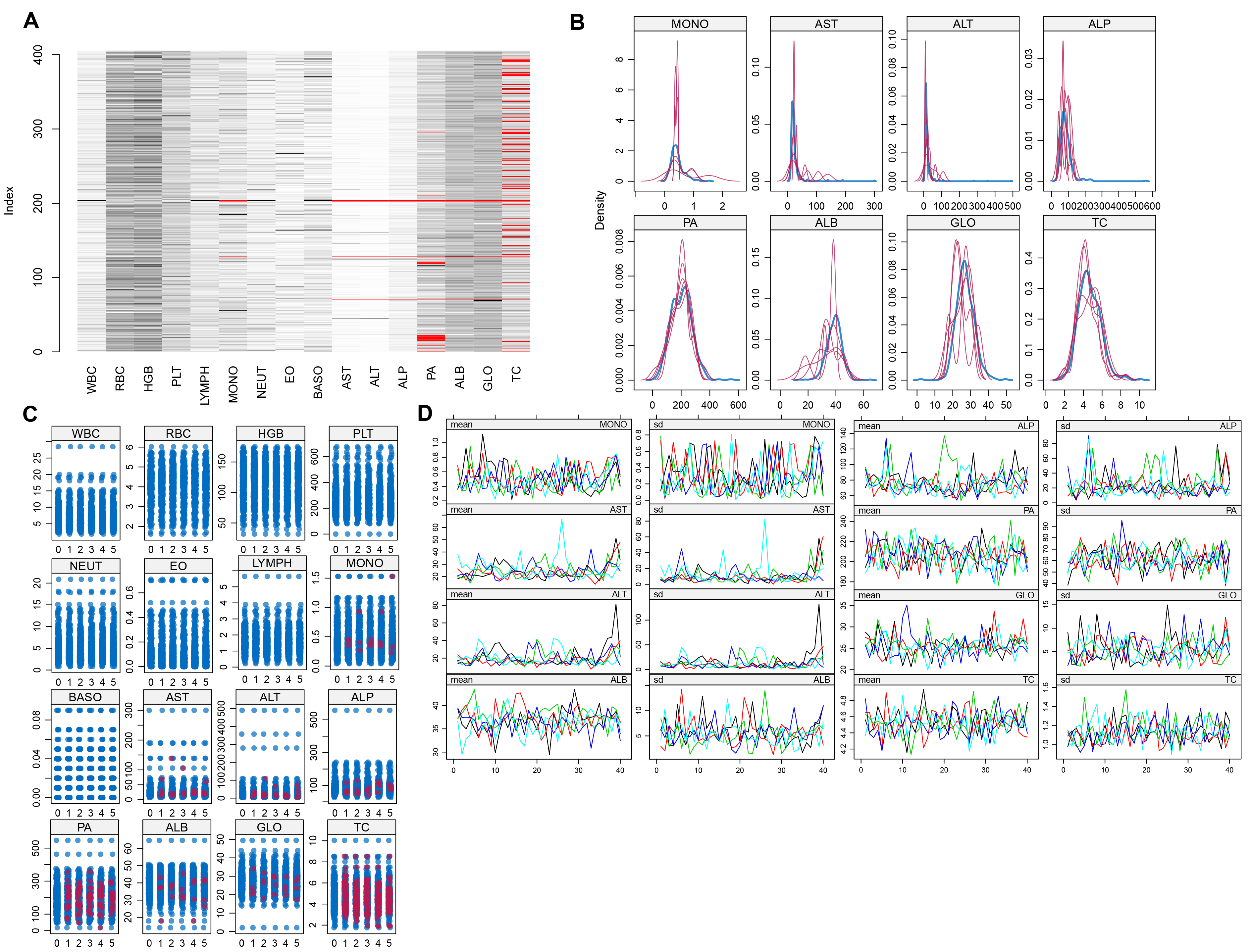

Supplement: Supplementary file 4 — Additional file 4: Figure S1. Missing values and multiple imputation. [file 12957_2022_2526_MOESM4_ESM.tif]

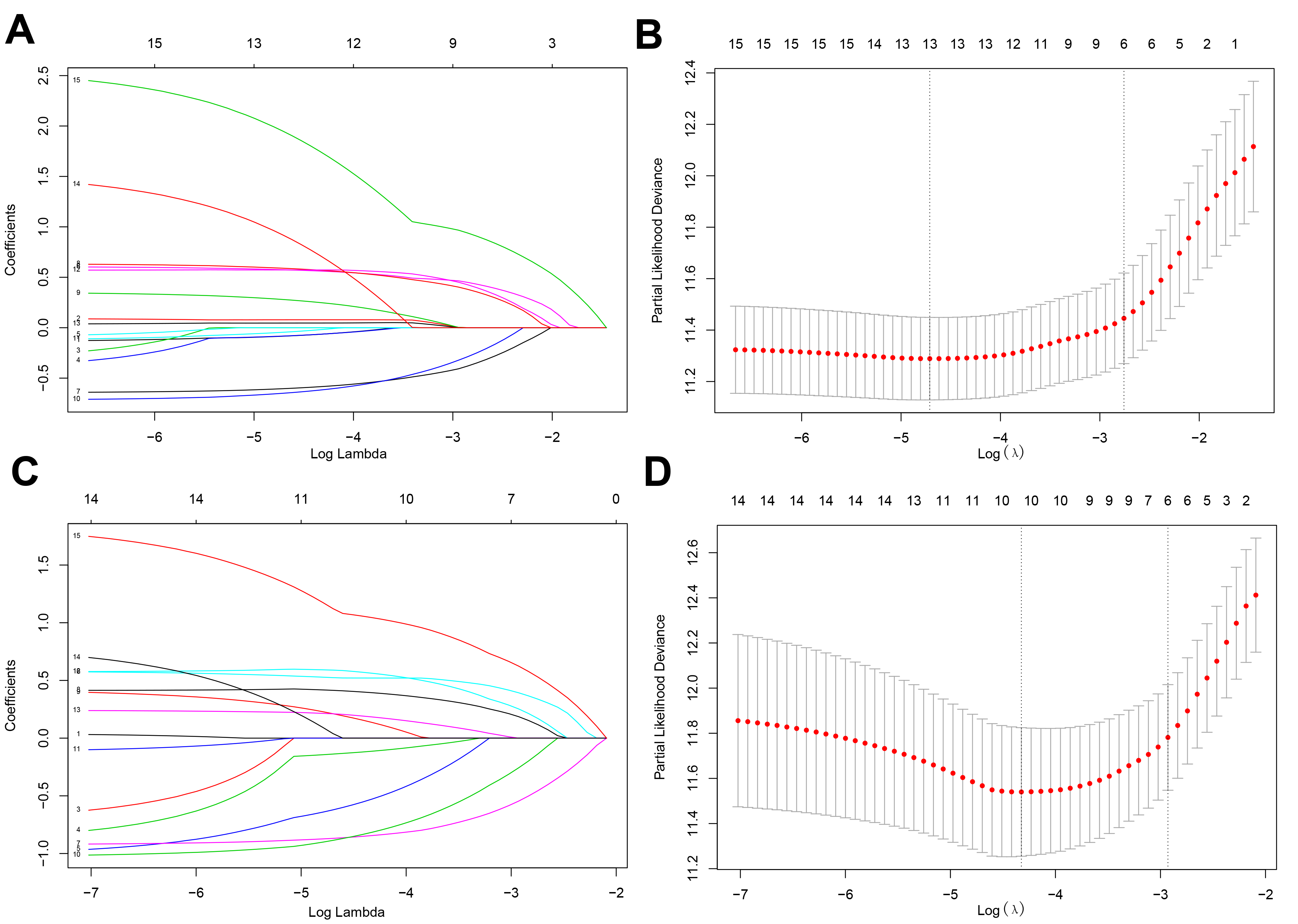

Supplement: Supplementary file 5 — Additional file 5: Figure S2. Clinical indicators selection using the LASSO Cox regression model. LASSO coefficients of total clinical indicators for PFS (A) and OS (C). Nonzero coefficients were determined based on the optimal log (lambda); log (lambda) and partial likelihood deviance were shown for PFS (B) and OS (D), the dotted line is displayed at the minimum log (lambda) represents the optimal number of predictors. [file 12957_2022_2526_MOESM5_ESM.tif]
